# Gut check: Unveiling the influence of acute exercise on the gut microbiota

**DOI:** 10.1113/EP091446

**Published:** 2023-09-13

**Authors:** Gregory J. Grosicki, Sean P. Langan, James R. Bagley, Andrew J. Galpin, Dan Garner, Jarrad T. Hampton‐Marcell, Jacob M. Allen, Austin T. Robinson

**Affiliations:** ^1^ Biodynamics and Human Performance Center Georgia Southern University GA USA; ^2^ Korey Stringer Institute, Department of Kinesiology University of Connecticut Storrs CT USA; ^3^ Muscle Physiology Laboratory San Francisco State University San Francisco CA USA; ^4^ Center for Sport Performance California State University, Fullerton Fullerton CA USA; ^5^ BioMolecular Athlete, LLC Wilmington DE USA; ^6^ Department of Biological Sciences University of Illinois at Chicago Chicago IL USA; ^7^ Department of Kinesiology and Community Health University of Illinois at Urbana‐Champaign Urbana IL; ^8^ Neurovascular Physiology Laboratory, School of Kinesiology Auburn University Auburn AL USA

**Keywords:** gut, human, microbiome, physical activity

## Abstract

The human gastrointestinal microbiota and its unique metabolites regulate a diverse array of physiological processes with substantial implications for human health and performance. Chronic exercise training positively modulates the gut microbiota and its metabolic output. The benefits of chronic exercise for the gut microbiota may be influenced by acute changes in microbial community structure and function that follow a single exercise bout (i.e., acute exercise). Thus, an improved understanding of changes in the gut microbiota that occur with acute exercise could aid in the development of evidence‐based exercise training strategies to target the gut microbiota more effectively. In this review, we provide a comprehensive summary of the existing literature on the acute and very short‐term (<3 weeks) exercise responses of the gut microbiota and faecal metabolites in humans. We conclude by highlighting gaps in the literature and providing recommendations for future research in this area.

## Abstract (highlights)

- **What is the topic of this review?**
  The chronic benefits of exercise for the gut microbiota are likely influenced by acute changes in microbial community structure and function that follow a single exercise bout. This review provides a summary of the existing literature on acute exercise responses of the gut microbiota and its metabolic output in humans.
- **What advances does it highlight?**
  Acute aerobic exercise appears to have limited effects on diversity of the gut microbiota, variable effects on specific microbial taxa, and numerous effects on the metabolic activity of gut microbes with possible implications for host health and performance.

## 1 INTRODUCTION

The human body is inhabited by a vast number of microorganisms living in complex ecological communities that influence human physiology (Dekaboruah et al., 2020). While these microorganisms, collectively known as the microbiota, are found in all body cavities and on human surfaces, the vast majority of the microbiota resides in the gastrointestinal tract (i.e., gut microbiota) (Sender et al., 2016). In 2007, the National Institutes of Health Common Fund Human Microbiome Project was established with the mission of generating resources to facilitate comprehensive characterization of the human microbiota and its role in human health (Turnbaugh et al., 2007). Subsequent findings have demonstrated that the health of the gut microbiota is critical in the maintenance of physiological homeostasis (Ding et al., 2019). For example, gut microbiota contributes to host health through the biosynthesis of vitamins and essential amino acids. Additionally, specific microbial taxa play a vital role in generating metabolic byproducts from undigested dietary components, such as the anaerobic fermentation of dietary fibre into short‐chain fatty acids (SCFAs) (Bäckhed et al., 2005). In contrast, gut microbiota dysbiosis is associated with the pathogenesis of intestinal disorders, such as inflammatory bowel disease (Singh et al., 2017), and extra‐intestinal disorders (Carding et al., 2015), including diabetes (Cani et al., 2007; Chen et al., 2021), Alzheimer's disease (Chen et al., 2022), cancer (Chaput et al., 2017) and cardiovascular disease (Koren et al., 2011). Importantly, the human gut microbiota demonstrates a high level of plasticity (Gomez et al., 2019), making it an attractive target for the prevention and treatment of disease.

Gut microbiota composition and function are sensitive to a variety of environmental factors (Krishnan et al., 2015; Lee et al., 2018). Health behaviours, including sleep (Grosicki et al., 2020), physical activity (Durk et al., 2019), sedentary behaviour (Bressa et al., 2017), hydration (Willis et al., 2021) and diet (Singh et al., 2017) have all been associated with alterations to the gut microbiota. Of these health behaviours, diet–microbiota interactions have received the most interest (Sonnenburg & Bäckhed, 2016; Torrey, 1919). Dietary manipulations, such as eating an all‐animal or all‐plant diet, rapidly (i.e., within 24 h) and reproducibly alter the composition of gut microbiota in humans (David et al., 2014).

More recently, exercise has emerged as a powerful modulator of both the composition and metabolic activity of the gut microbiota (Monda et al., 2017). For instance, aerobic exercise induces compositional and functional changes in the human gut microbiota that are independent of diet (Allen et al., 2018), and are reversed with training cessation (Allen et al., 2018; Hampton‐Marcell et al., 2020). The effects of chronic exercise training on the gut microbiota have been extensively reviewed (Clauss et al., 2021; Dziewiecka et al., 2022; Mailing et al., 2019; Monda et al., 2017; Ramos et al., 2022). Hallmark microbial benefits of chronic exercise participation include: (i) increased SCFA production, (ii) improved gut epithelial integrity, (iii) increased mucin‐degrading bacteria, and (iv) increased capacity for energy harvest. Notably, the benefits of chronic exercise for the gut microbiota are likely influenced by acute changes in microbiota structure and function that follow a single exercise bout (i.e., acute exercise). However, there is limited information on the effects of acute exercise on gut microbiota. Importantly, an improved understanding of changes in the gut microbiota that occur with acute exercise could aid in the development of evidence‐based exercise training interventions to more effectively target the gut microbiota to improve health and performance. Therefore, in this review, we summarize existing literature that has characterized acute and very short‐term (<3 weeks) exercise responses of the gut microbiota and faecal metabolites in humans. We conclude by highlighting critical gaps in the literature and providing recommendations for future research in this area.

## 2 INFLUENCE OF ACUTE EXERCISE ON THE GUT MICROBIOTA

A literature search was carried out using PubMed and Web of Science between December 2022 and July 2023 for all studies (not date restricted) published in English combining the terms ‘gut microbiome’ OR ‘gut microbiota’ AND ‘acute exercise’ OR ‘short‐term exercise’. References listed in original papers and reviews were examined and a literature search was conducted by following up on references quoted in relevant articles.

As of 1 July 2023, we identified 11 studies that characterized changes in the gut microbiota following acute and very short‐term (<3 weeks) exercise in human participants (*n* = 191; 147 males and 54 females, Table 1). Below, we summarize findings from these studies regarding the effects of acute exercise on: (i) intra‐ (α) and inter‐individual (β) diversity, (ii) the relative abundance of specific microbial taxa and activity of metabolic pathways, and (iii) faecal metabolites.

**TABLE 1:** Studies examining acute exercise responses of the gut microbiota and faecal metabolites in humans.

							Findings		
References	Population	Exercise stimulus	Time points	Non‐exercising control (Y/N)	Dietary control (Y/N)	Microbiome measures	Diversity	Individual taxa and metabolic pathways	Metabolites
Shukla et al. (2015)	*n* = 20 10 ME/CFS 10 controls	Cycling $\dot{V}O_{2\;\max}$	•Pre (not specified) •Post (∼72 h)	N	N	16S rRNA	N/R	• Taxa: ↑ 7 of 9 phyla in ME/CFS vs. 2 of 9 in Controls ↑ blood levels of bacterial phyla in ME/CFS vs. Controls • Pathways: N/R	N/R
Karl et al. (2017)	*n* = 71M/2F Soldiers >18 years, ∼24 kg/m^2^	Four‐day, 51‐km XC Ski with 45 kg pack	• Pre (≤2 days) • Post (night/day after)	N	Y • Control group: Normal diet • PRO group: Normal + PRO (∼150 g/day) • CHO group: Normal + CHO (∼450 g/day)	16S rRNA + untargeted metabolomics	• α‐Diversity: ↑ Shannon ↔ Chao1 and OTUs • β‐Diversity: Δ sig, independent of diet	• Taxa: Δ > 50% of genera; ↑ less dominant genera and ↓ *Bacteroides* • Pathways: N/R	• Δ 274 compounds: ↓ metabolites of AA, FA, CHO, energy metabolism, BA; ↑ *p*‐Cresol • Δ microbiota explained 23% of Δ metabolites
Zhao et al. (2018)	*n* = 16M/4F Amateur runners 31 ± 6 years, 22 ± 2 kg/m^2^	Half‐marathon	• Pre and post (not specified)	N	Measured, not controlled	16S rDNA + untargeted metabolomics	• α‐Diversity: ↔ • β‐Diversity: N/R	• Taxa: Δ 15 OTUs – ↑ in 7; ↓ in 8 • Pathways: 23 KEGG pathways; ↑ flagellar assembly, bacterial chemotaxis, and cell motility; ↓ oxidative phosphorylation	• Δ 40 metabolites: mainly nucleic and organic acids • 26 associations between Δ metabolites (*n* = 19) and Δ taxa (*n* = 4); increase in *Coriobacteriaceae* associated with 15 metabolites
Grosicki et al. (2019)	*n* = 1M World‐class runner 32 years, 15% bf	161‐km mountain footrace	• Pre: 21 weeks • Pre: 2 weeks • Post: 2 h • Post: 10 days	N	N	16S rRNA	Pre vs. Post (2 h)		
							• α‐Diversity: ↑ Shannon • β‐Diversity: N/R	• Taxa: Δ several genera –↑*Veillonella* and *Streptococcus;* ↓ *Alloprevotella* and *Subdolingranulum* • Pathways: N/R	N/R
Scheiman et al. (2019)	*n* = 25 15 runners 10 controls	Marathon	• Pre: 6‐day samples • Post: 5‐day samples	Y	N	16S rDNA	N/R	• Taxa: *Veillonella* most differentially abundant (↑); reproduced with shotgun seq in runners and rowers (*n* = 87) • Pathways: ↑ methylmalonyl‐CoA (lactate→SCFAs)	NR
Keohane et al. (2019)	*n* = 4M Rowers 26 ± 1 years, 18 ± 8% bf	33‐day Transoceanic row 2 h on/2 h off	• Pre • Mid (day 17) • Post (day 33) • Recovery (3‐months)	N	Measured, not controlled	Shotgun	Pre vs. Mid (Day 17)		
							• α‐Diversity: ↑ Shannon: 3 of 4 • β‐Diversity: N/R	• Taxa: Δ some species—↑ butyrate producers: *Dorea longicatena*, *Rosebruia hominis*, *Subdolongranulum*; ↓ *Bacteroides finegoldii* • Metabolic pathways: ↑ AA and FA production	NR
Tabone et al. (2021)	*n* = 40M XC runners 36 ± 8 years, 23 ± 2 kg/m^2^	$\dot{V}O_{2\;\max}$ + 1‐km max run	• Pre (morning) • Post (≤4 h)	N	Measured, not controlled	16S rRNA + untargeted metabolomics	• α‐Diversity: ↔ Shannon, Faith, and OTUs • β‐Diversity: ↔ Bray–Curtis, Jaccard and UniFrac	• Taxa: Δ 6 taxa: *Romboutsia*, *Escherichia coli* TOP498, *Ruminococcaceae* UCG‐005, *Blautia*, *Ruminoclosterium 9*, and *Clostridium phoceensis* • Pathways: ↑ Phenylalanine, tyrosine and tryptophan biosynthesis	• Δ 12 metabolites: Tryptohan, Methionine, *S*‐ethyl‐l‐cysteine, phenol, 4‐hydroxyl‐alcohol, l/d‐glutamine, serine, phenylalanine, tyrosin, hexose alcohols, pyridoxamine, cinnamic acid • *Ruminiclostridium* 9 most associated with metabolites
Oliveira et al. (2022)	*n* = 21F Elite footballers 24 ± 3 years, 17 ± 5% bf	10 day tournament	• Pre (day 2–3) • Post (day 9–10)	N	Measured, not controlled	16S rRNA	• α‐Diversity: ↔ Shannon • β‐Diversity ↔ Bray–Curtis	• Taxa: No Δ in any bacterial species • Pathways: N/R	N/R
Fukuchi et al. (2022)	*n* = 9M/4F 19–21 years, ∼20 kg/m^2^	5 km (males), 3 km (females)	• Pre and post (not specified)	N	Measured, not controlled crossover study (Control vs. fermented soymilk)	16S rRNA	Control Period		
							• α‐Diversity: ↓ Shannon ↔ Chao1 • β‐Diversity Δ sig, UniFrac	• Taxa: Δ phyla: ↓ *Bacteroidetes* and ↑ *Firmictutes* • Pathways: N/R	• Δ 27 urinary metabolites • Relations between gut microbiota and urinary metabolites, e.g., *Firmicutes* correlated with tricarballylic acid and carboxylic acid
Sato and Suzuki (2022)	*n* = 9M 47 ± 6 years, 11 ± 3% bf	∼100 km footrace	• Pre (≤72 h) • Post (first bowel movement) • Recovery (∼10 days)	N	Measured, not controlled	16s rRNA	• α‐Diversity: ↔ ACE, Chao1, Fisher, observed, Shannon, Simpson • β‐Diversity: ↔	• Taxa: Δ species: ↑ *Collinsella aerofaciens*, *Catenibacillus scindens*, *Clostridium* sp. E2, *Ruminococcaceae* bacterium LM158, *Alistipes putredinis*, *Drancourtella massiliensis*, *Massiliomicrobiota timonensis*, *Clostridium* sp. Culture Jar 17, *Romboutsia timonensis*, and *Streptococcus sanguinis;* ↓ *Blautia luti*, *Eubacterium rectale*, *Faecalibacterium prausnitzii* • Pathways: N/R	N/R
Grosicki, Pugh et al. (2023)	*n* = 9M/3F 43 ± 14 years, 23 ± 2 kg/m^2^	Ironman triathlon	• Pre (≤48 h) • Post (16 ± 13 h)	N	Measured, not controlled	Shotgun + targeted metabolomics	• α‐Diversity: ↔ Shannon, Simpson and OTUs • β‐Diversity: ↔ Bray–Curtis and Jaccard	• Taxa: No Δ in any bacterial species • Pathways: N/R	• ↓ Primary and secondary bile acids • ↑ Long‐chain fatty acids • ↓ Short‐chain fatty acids

AA, amino acids; Ace, Abundance‐based Coverage Estimator; BA, bile acids; CHO, carbohydrate; FA, fatty acids; ME/CFS, myalgic encephalomyelitis/chronic fatigue syndrome; N/R, not reported; OTU, operational taxonomic unit; PRO, protein; XC, cross‐country; % bf, percent body fat.

### 2.1 Intra‐ and inter‐individual diversity

#### 2.1.1 Effects of acute exercise on α‐diversity

Intra‐individual (α‐diversity) summarizes the structure of an ecological community with respect to its richness (number of dominant taxonomic groups), evenness (distribution of abundances of the groups) or both (Willis, 2019). Microbial diversity facilitates host resilience through a variety of mechanisms including immunity, pathogen protection and energy metabolism. Thus, a more diverse microbiota is generally considered a healthier microbiota (Deng et al., 2019), although this is not always the case (Sanapareddy et al., 2012). Additionally, conditions such as obesity (Ley et al., 2005), inflammatory bowel disease (Qin et al., 2010) and type 2 diabetes (Chen et al., 2021) are typically associated with lower microbial diversity.

We identified nine studies that evaluated α‐diversity of the gut microbiota before and after an acute exercise bout in human participants. Five of the nine studies reported no differences (Grosicki, Pugh, et al., 2023; Oliveira et al., 2022; Sato & Suzuki, 2022; Tabone et al., 2021; Zhao et al., 2018), which is consistent with observations from 6‐ to 8‐week training studies in humans (Allen et al., 2018; Cronin et al., 2018) and mice (Lamoureux et al., 2017) reporting no changes in α‐diversity. However, cross‐sectional investigations report greater α‐diversity in athletes and physically active individuals compared with sedentary controls (Clarke et al., 2014). Moreover, α‐diversity is associated with cardiorespiratory fitness in healthy individuals irrespective of body composition and dietary habits (Estaki et al., 2016). Consistent with these findings, we identified three studies demonstrating increased post‐exercise α‐diversity (Grosicki et al., 2019; Karl et al., 2017; Keohane et al., 2019), though two of these studies did not perform hypothesis testing due to insufficient sample sizes (*n* = 1 and 4). It is important to note that a common similarity of these three studies is that they all investigated acute microbiota responses to rather extreme exercise stimuli (i.e., ultra‐marathon, transoceanic row, and a 4‐day military cross‐country ski), and that increased α‐diversity was detected using the Shannon Index, which considers both the richness and evenness of taxa. Post‐exercise changes in α‐diversity have yet to be detected using other diversity metrics (e.g., Simpson, Chao1 and Faith). Taken together, these findings suggest that moderate acute and short‐term exercise appear to have limited effects on microbial diversity. Extreme exercise (e.g., ultra‐marathons), may induce transient changes to α‐diversity (via Shannon Index), but these findings need to be verified by additional controlled studies across larger and more diverse populations.

#### 2.1.2 Effects of acute exercise on β‐diversity

Inter‐individual diversity (β‐diversity) describes the species diversity between two (or more) microbial communities (e.g., microbial communities before and after exercise). β‐Diversity is determined by statistical techniques that quantify the distance between microbiota pairs, which can be evaluated using distance‐based hypothesis testing (e.g., permutational multivariate analysis of variance) (Kelly et al., 2015). Of the 11 studies that investigated the effects of acute exercise on the gut microbiota, only six included β‐diversity analyses, four of which reported no changes (Grosicki, Pugh, et al., 2023; Oliveira et al., 2022; Sato & Suzuki, 2022; Tabone et al., 2021) and two reported changes (Fukuchi et al., 2022; Karl et al., 2017). In our view, no immediate, distinguishable differences between these studies is apparent. Given these limited, mixed findings, it would be presumptive to make any definitive conclusions regarding the effects of acute exercise on β‐diversity. Moreover, β‐diversity findings from short term (i.e., 6–8 weeks) exercise training studies in humans have similarly produced mixed results (Allen et al., 2018; Cronin et al., 2018). Clearly, more work featuring greater experimental control (discussed in detail below; see section 3, ‘Gaps in knowledge and future directions’) is needed to delineate the effects of acute exercise on β‐diversity.

### 2.2 Effects of acute exercise on microbial taxa and metabolic potential

While diversity analyses reflect overall patterns in microbiota variation, a limitation of these techniques is that they may miss subtle changes in low‐abundance microbiota that may play important roles in host physiology (Su, 2021). To capture these changes, investigations have performed pairwise comparisons of amplicon sequence variants (ASVs), ranging from the phylum (Shukla et al., 2015) to the species level (Grosicki, Pugh, et al., 2023; Oliveira et al., 2022; Sato & Suzuki, 2022; Scheiman et al., 2019; Tabone et al., 2021). Of the 11 studies included in this review, nine reported changes in specific microbial taxa (Fukuchi et al., 2022; Grosicki et al., 2019; Karl et al., 2017; Keohane et al., 2019; Sato & Suzuki, 2022; Scheiman et al., 2019; Shukla et al., 2015; Tabone et al., 2021; Zhao et al., 2018). The most cited of these studies was published in 2019 by Scheiman et al., who reported an increased relative abundance of *Veillonella* in 15 runners following participation in the Boston marathon (Scheiman et al., 2019). Importantly, the rigor of these findings was strengthened by comparison to a non‐exercising control group (*n* = 10), as well as replication of post‐exercise increases in *Veillonella* relative abundance in ultra‐marathon runners and Olympic trail rowers (*n* = 87). These findings prompted subsequent experiments in rodents suggesting that post‐exercise increases in *Veillonella*, and particularly *Veillonella atypica*, may benefit host performance via metabolic conversion of exercise‐induced lactate into propionate. Intriguingly, our group observed a striking 143‐fold increase in the relative abundance of *Veillonella* in a world‐class ultramarathon runner ∼2 h following participation in the 161‐km Western States Endurance Run (Grosicki et al., 2019). However, post‐exercise increases in *Veillonella* were not reported in 9 of 11 identified acute exercise microbiota studies. These divergent findings indicate interindividual differences in microbiota responses to acute exercise and highlight a need for research into what factors contribute to this heterogeneity. For example, training status has been identified as an important consideration for the nature of responses to an exercise stimulus (Egan & Sharples, 2023), and may explain selective increases in *Veillonella* relative abundance in highly trained athletes (i.e., Boston Marathon runners, Olympic rowers and a world‐class ultramarathon runner).

Two investigations of acute exercise on microbiota responses (Grosicki, Pugh, et al., 2023; Oliveira et al., 2022), including our own (Grosicki, Pugh, et al., 2023), demonstrated no change in any bacterial species post‐exercise. While in contrast to many studies in this area, stability and resilience have been identified as essential ecological characteristics of the gut microbiota with relevance for human health (Fassarella et al., 2021). Thus, despite limited data, it seems justifiable that acute exercise may have limited effects on microbial community structure, particularly in highly fit individuals who are accustomed to habitual exercise. Inspection of possible explanations for these inter‐study differences reveals a key limitation in the existing acute exercise microbiota literature. Specifically, only 2 of 11 studies, including our recent work demonstrating no changes in any bacterial species following an Ironman triathlon (Grosicki, Pugh, et al., 2023), implemented whole genome shotgun sequencing. Importantly, whole genome shotgun sequencing has multiple advantages over the 16S amplicon method including enhanced resolution and accurate identification of bacterial species as well as increased detection of diversity and prediction of genes (Ranjan et al., 2016). The only other study to use shotgun sequencing reported an increase in some butyrate‐producing species (Keohane et al., 2019), though the sample size was modest (*n* = 4) and the stimulus was exceptionally greater (i.e., transoceanic row) than most other studies in the present review. It is also worth noting that both studies demonstrating no post‐exercise differences in bacterial taxa when analyses were performed at the species level. It may be that small and acute changes in bacterial species post‐exercise summate to yield meaningful shifts in higher‐level microbial taxa that are more readily apparent at the phylum (Fukuchi et al., 2022; Shukla et al., 2015) or genus level (Karl et al., 2017).

**FIGURE 1:**
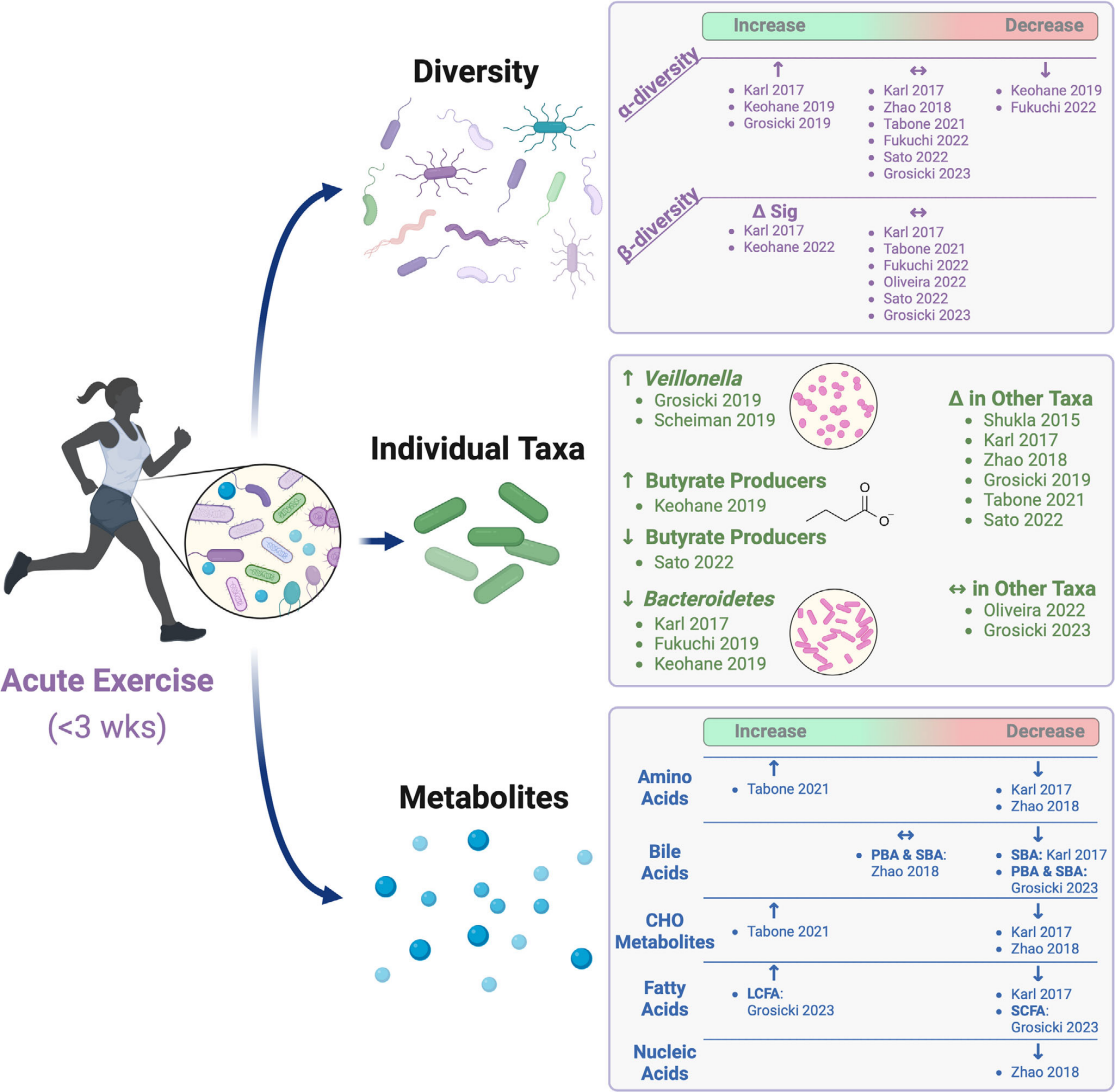
Summary of findings on gut microbiota changes with acute exercise. We identified 11 studies characterizing changes in gut microbiota following acute and very short‐term (<3 weeks) exercise in human participants. As depicted, these studies suggest that acute aerobic exercise has: (i) limited effects on intra‐ (α) and inter‐individual (β) diversity of the gut microbiota that may be more distinguishable with acute bouts of prolonged/extreme exercise, (ii) variable effects on specific microbial taxa that are likely a function of heterogeneity in exercise stimuli and interindividual factors, as well as methodological differences for microbiota measurements, and (iii) numerous effects on the metabolic activity of gut microbes with possible implications for host health and performance. CHO, carbohydrate; LCFA, long‐chain fatty acids; PBA, primary bile acids; SBA, secondary bile acids; SCFA, short‐chain fatty acids. Created with BioRender.com.

Beyond taxonomic comparisons, some investigations of acute exercise and the gut microbiota have also sought to characterize changes in the metabolic activity of the gut microbiota. For instance, Zhao et al. (2018) identified 23 Kyoto Encyclopedia of Genes and Genomes (KEGG) pathways, inclusive of those associated with flagellar assembly, bacterial chemotaxis, and cell motility, that were significantly altered by running a half‐marathon. There also seems to be an upregulation of pathways associated with energy metabolism, as evidenced by the upregulation in the methylmalonyl‐CoA pathway (lactate → propionate and acetate) (Scheiman et al., 2019). Additionally, there appears to be an increase in the activity of pathways associated with amino acid and fatty acid production (Keohane et al., 2019; Tabone et al., 2021). These preliminary findings are depicted in Figure 1 and allude to the importance of acute changes in the metabolic activity of the gut microbiota during exercise as an important mechanism through which increased host energy requirements are satisfied. However, there is still much to learn regarding the effects of acute exercise on gut microbiota metabolic pathway activity, including the persistence of these changes as well as their relations to host health and performance.

### 2.3 Effects of acute exercise on microbial‐derived metabolites

Microbial metabolites are bioactive and have considerable effects on human physiology. Thus, the gut microbiota may be best assessed by directly analysing its metabolic output (alongside multi‐omics data integration), which provides a more complete picture of gut microbial ecology and metabolism (Knight et al., 2018). While most directly quantified in faeces, we acknowledge that microbial metabolites may also be detected in blood, and that exercise drives acute changes in blood xenometabolites (i.e., ‘non‐self’ molecules, from microbes or food) (Grapov et al., 2019). However, the origin (e.g., gastrointestinal microbes, adipose tissue pools, etc.) of these metabolites is not always easy to identify, and many of the blood metabolites influenced by exercise remain to be annotated. Given the need for more work in this area, in the present work we focus solely on metabolite changes (or lack thereof) in stool samples.

We identified four studies characterizing the acute effects of exercise on faecal metabolites, all of which reported substantial pre–post differences. The only study we identified that included a dietary control group reported changes in 274 compounds in faecal samples after a 4‐day cross‐country ski‐march, most of which represent reductions in metabolites of amino acid, fatty acid, carbohydrate and energy metabolism, as well as secondary bile acids (Karl et al., 2017). Interestingly, the study reported an increase in *p*‐cresol sulfate, a metabolite of tyrosine fermentation that is associated with the progression of chronic kidney disease (Liu et al., 2018) and cardiovascular risk in haemodialysis patients (Meijers et al., 2008; Shafi et al., 2015). However, it is unclear whether this could simply be a hormetic response that elicits adaptation. In contrast, shorter duration acute exercise (i.e., less than multiple hours) seems to induce fewer changes in faecal metabolites, which serves as evidence of a possible dose–response relation between acute exercise and metabolite alterations. For instance, a half‐marathon (∼2 h) resulted in changes in 40 metabolites (i.e., increases in organic acids and reductions in nucleic acids) (Zhao et al., 2018), whereas a much shorter duration exercise bout (i.e., $\dot{V}O_{2\;\max}$ test and 1‐km maximal effort run) resulted in alterations in only 12 metabolites (Tabone et al., 2021). It remains to be determined whether exercise duration or exercise volume (i.e., duration × intensity) is the primary driver of the apparent dose‐dependency between exercise and acute faecal metabolite changes. Preliminary insight into the effects of exercise volume on the gut microbiota is provided by a recent study of highly trained cross country athletes (*n* = 14) with serial measurements following 3 weeks of normal training, 3 weeks of high‐volume training and following a 1‐week taper; changes in microbial taxa were detected following high‐volume training and these alterations persisted after a taper (Craven et al., 2022). Notably, all of the aforementioned studies observed associations between changes in bacterial taxa and metabolites, which supports the likelihood of a causal link between these alterations.

Recently, we conducted the first study to implement targeted metabolomics analysis of faecal samples following acute exercise, with a focus on bile acids and fatty acids as different functional readouts of the gut microbiota that are demonstrated to be responsive to exercise. Following participation in an Ironman triathlon, we observed a global decrease in faecal bile acids (primary and secondary) and SCFAs (Grosicki, Pugh, et al., 2023), which is in agreement with previous reports (Karl et al., 2017). However, we observed an increase in the long‐chain fatty acids oleic acid and palmitoleic acid following race completion, which we speculated may have been an adaptive response to increased whole body (i.e., host) oxygen consumption and fatty acid oxidation throughout the race, though poor absorption owing to gastrointestinal stress may have contributed. Collectively, these studies align to support a potent influence of acute exercise on the faecal metabolome, with changes that seem to be proportional to the metabolic demand of the exercise bout.

## 3 GAPS IN KNOWLEDGE AND FUTURE DIRECTIONS

Acute exercise appears to modestly impact microbial composition and significantly modify its metabolic output. Yet, a lack of rigorous experimental control has left a lack of consensus as to what specific microbial taxa and metabolites change in response to acute exercise. To rectify this uncertainty, we recommend that future studies consider the following when examining the effects of acute exercise on the gut microbiota: (i) study design controls, (ii) dietary controls, (iii) detailed reporting of participant characteristics and attention to relevant biological (e.g., sex) and sociocultural (e.g., race and ethnicity) variables, (iv) evaluation of the impact of different exercise modes and doses, as well as environmental conditions on acute microbiome responses, and (v) a (reverse) translational approach considering microbiome transplantation and/or bioreactors.

### 3.1 Study design controls

To date, most of the studies on acute exercise and gut microbiota have been field studies (Table 1). While the novelty and ecological validity of such studies is admirable, there is a clear need for more rigorous, well‐controlled laboratory‐based investigations of microbiota responses to acute exercise. Only one of 11 identified acute exercise microbiota studies included a non‐exercising control group (Scheiman et al., 2019). This limitation is particularly problematic in the context of temporal variability in human gut microbiota profiles, inclusive of substantial day‐to‐day fluctuations in diversity and species evenness (Vandeputte et al., 2021). To mitigate this confound, future work in this area should seek to include a non‐exercise control group or employ a within‐subjects crossover design (i.e., exercise day vs. non‐exercise day). Additionally, substantial inter‐individual variability in gut microbiota profiles and host responses makes it challenging to predict individual responses to a given intervention (Healey et al., 2017). As a result, statistical techniques such as mixed‐effects models to control for interindividual variation or analyses that consider microbial variation in lower dimensions (e.g., t‐SNE) may be necessary.

A unique challenge in the field of microbiota research is that unlike other biospecimens, samples cannot be collected at a specific, predetermined time point. Thus, capturing acute changes in microbial ecology, such as those that might occur following a single exercise bout, can be challenging. To alleviate this issue, future studies should seek to include a larger number of participants and are encouraged to consider collecting microbiota measures on more than one sample per time point (e.g., daily samples leading into and following the exercise bout). Careful documentation of date and time of sample collection will bolster the rigor of such studies, and may aid in the delineation of a time course for gut microbiota changes following exercise. Importantly, bacterial community composition and faecal metabolite profiles (e.g., SCFAs) are affected by collection methods (Jones et al., 2021). Immediate freezing without preservative is widely considered the gold standard for gut microbiome analyses, as this method preserves microbial composition similar to analysis of a fresh sample (Wang et al., 2018). While alternative collection methods, such as at‐home collection kits used by existing field studies, produce results that are generally comparable to immediate freezing, differences in certain measures of gut microbiota and faecal metabolites haven been reported (Wang et al., 2018). Thus, future laboratory‐based studies are encouraged to employ immediate freezing of collected specimens when possible, consistent with contemporary best practice (Jones et al., 2021).

Acquisition and measurement of xenometabolites in participant blood samples is another method that may be used to overcome limitations associated with the collection of stool samples at imprecise time points. As mentioned above, acute exercise is reported to increase several microbially derived xenometabolites (Grapov et al., 2019). Recent findings suggest that 6 weeks of aerobic exercise training modifies xenometabolites in the gut and circulation of lean and obese humans in a physiologically relevant manner (Kasperek et al., 2023). Regarding very short‐term exercise, several significant changes in plasma concentrations of metabolites known to be partially or fully derived from microbial metabolism were reported following a 4‐day cross‐country ski march (Karl et al., 2017). Future studies are encouraged to build on these approaches to provide greater insight into how acute exercise‐induced changes to xenometabolite production and absorption impact host physiology.

### 3.2 Dietary control

There is evidence to suggest that diet may be a stronger determinant of microbiota composition than exercise (Cronin et al., 2018; Yun et al., 2022). While many acute exercise microbiota studies characterized dietary intake (Table 1), only one implemented a dietary control (Karl et al., 2017). Importantly, in mice, diet and exercise orthogonally alter the gut microbiota (Kang et al., 2014), highlighting the unique impact of these different lifestyle behaviours on microbial community structure. Specifically, both a high fat diet (relative to normal chow) and exercise led to large changes in gut microbiota but with little overlap in specific taxa that were affected (Kang et al., 2014). To delineate the effects of exercise alone on the gut microbiota, future studies are encouraged to control and standardize dietary intake for at least 3 days leading into exercise and throughout the study duration (Allen et al., 2018; David et al., 2014), inclusive of any food or liquid consumed during an exercise bout. Given relations between hydration and the gut microbiota it may also be important to control and standardize fluid intake (Vanhaecke et al., 2022; Willis et al., 2021), though interventional studies demonstrating a causal role for hydration status in influencing the gut microbiota are needed.

### 3.3 Reporting of participant characteristics

As with all human subject research, detailed reporting of participant characteristics for exercise microbiome research is essential. For example, sex, age, weight and underlying health conditions may affect study outcomes and have been consistently reported across acute exercise gut microbiota studies. However, likely due to its relative infancy, existing acute exercise and gut microbiota literature has failed to account for the influence of potentially relevant social factors (e.g., race and ethnicity) that may influence human health, the microbiota and microbiota responses to acute exercise (Grosicki et al., 2022, Grosicki, Flatt, et al., 2023; Robinson et al., 2022). Indeed, racial differences in acute physiological responses to exercise have been reported (Schroeder et al., 2019), but whether there are racial differences in responses and/or adaptations of the gut microbiota to exercise is unclear and deserving of future inquiry. It is also possible that the gut microbiota may fluctuate based on menstrual phase and/or due to contraceptive use (Mihajlovic et al., 2021), and thus these variables should be considered when designing and reporting on findings from exercise gut microbiota research.

The relative novelty of the acute exercise stimulus (i.e., initial or repeated exposure) as well as the training status of the participants is another important consideration for studies going forward. Indeed, previous exposure to an exercise stimulus may impact subsequent acute physiological and perceptual responses (Lincoln et al., 2022), and a recently published systematic review noted that training status may impact gastrointestinal responses to aerobic exercise, with greater plasma indicators of intestinal permeability/distress following acute exercise in untrained compared with trained individuals (Bonomini‐Gnutzmann et al., 2022). More work is needed to determine whether these findings are recapitulated through greater resiliency of the gut microbiota in habitual exercisers compared with sedentary individuals.

### 3.4 Impact of exercise mode, dose and environmental conditions on acute microbiome responses

To the best of our knowledge, there has not been a single study to date to evaluate microbiota responses to acute resistance exercise. Moreover, evidence regarding the influence of chronic resistance training on the gut microbiota is also scarce. Preliminary data in humans (Bycura et al., 2021; Moitinho‐Silva et al., 2021) and rodents (Fernández et al., 2021) suggest that short‐term and chronic (i.e., 4–8 weeks) aerobic and resistance exercise training may differentially affect gut microbiota structure, with fewer microbial benefits detected following resistance training (Bonomini‐Gnutzmann et al., 2022). Given the immense health benefits conferred by resistance exercise and its growing popularity (Westcott, 2012), there is strong impetus for future studies seeking to improve understanding of gut microbial responses and adaptations to acute and chronic resistance exercise. Lastly, while existing studies have explored gut microbiota responses to aerobic exercise bouts of various intensities and durations, none of these studies have compared gut microbiota responses to different bouts of exercise in the same population. Acute and chronic physiological responses to exercise vary substantially as a function of exercise intensity and duration, and it seems likely that intensity‐ and duration‐dependent exercise‐induced splanchnic hypoperfusion may contribute to differential remodelling of gut microbial ecology (van Wijck et al., 2011). A more definitive understanding of how classic exercise‐related variables (i.e., mode, intensity, and duration) influence gut microbiota responses to acute and chronic exercise may aid in the development of specialized, microbiota‐targeted exercise strategies to improve health and performance.

In addition to exercise modality, intensity and volume, environmental conditions (e.g., temperature, humidity and/or altitude) can substantially alter acute physiological responses to exercise. The recent trend for global climate change further underscores the importance of understanding how environment influences exercise responses (Kenney et al., 2022), but the influence of these factors on gut microbiota responses to acute exercise is uncertain. For example, hyperthermia‐related gastrointestinal dysfunction (Hall et al., 2001) and heat‐induced gut injury may be involved in heat stroke pathophysiology (Bouchama et al., 2022). Whether the gut microbiota modulates this response is unclear, but there are documented associations between pre‐exercise α‐diversity and post‐exercise cytokinaemia, as well as core temperature, intestinal permeability, and specific bacterial taxa following 2 h of exercise heat stress (Bennett et al., 2020). Additionally, the microbiota contributes to metabolic heat production, further extending the connection between the gut and thermoregulation (Armstrong et al., 2019). It is tempting to speculate this could be a possible mechanism through which the gut microbiota may influence health and performance.

Indeed, there are reports that have associated microbiota alterations after heat acclimation to reduced organ damage biomarkers (Liu et al., 2023). However, to date, no studies have obtained acute pre‐ and post‐exercise samples with heat stress. Nonetheless gut microbiota are responsive to changes in environmental pressure (Kleessen et al., 2005; Li & Zhao, 2015; Li et al., 2016), and such changes may be associated with altered immunological challenge and health risk (Kleessen et al., 2005). In mice, exposure to 4 weeks of hypoxia induced gut microbiota changes characterized by increased abundance of *Akkermansia* and *Bacteroidetes* genera and related SCFAs in a manner that was causally linked to improvements in endurance performance (Huang et al., 2021). In consideration of these studies, we speculate that environmental conditions (i.e., temperature and altitude) influence gut microbiota responses to acute exercise. A deeper understanding of the interaction between acute exercise, environmental exposures and the gut microbiota may aid in developing strategies to potentiate the benefits of exercise for the gut microbiota and host function via manipulation of exercise environment.

### 3.5 A (reverse) translational approach

Translational research involves a ‘bench‐to‐bedside’ approach whereby knowledge from basic sciences is harnessed to produce new drugs, devices and treatment options for patients (Woolf, 2008). In the context of human gut microbiota and exercise research, reversal of this approach through animal experiments in highly controlled environmental conditions can be used to test causal relations between the gut microbiota and host phenotype (Ericsson & Franklin, 2015). Making use of this model, Scheiman et al. implemented a treatment/control crossover trial in mice demonstrating that *Veillonella atypica* directly isolated from human runners extends treadmill run time to exhaustion when compared with a control (*Lactobacillus bulgaricus*). While no other acute exercise gut microbiota studies to date have used such rigorous experiments, a recent study in genetically and metagenomically diverse mice used broad‐spectrum antibiotics (i.e., microbiota ablation) to uncover the impact of the gut microbiota on brain circuits involved in exercise performance (Dohnalová et al., 2022). Specifically, microbiota depletion blunted the exercise‐induced surge in dopamine, which may have implications for exercise motivation and physical performance. Studies such as this, and others (Liu et al., 2020), highlight the immense value of human‐to‐animal microbiota transplantation as a means to confirm the causal role of the gut microbiota on host phenotype.

Ex vivo, culture‐based approaches provide a direct lens through which to understand the charactersitics features and phenotypes of specific bacteria. However, most intestinal bacteria are considered ‘unculturable’, and those that are require highly advanced techniques (Ito et al., 2019). Moreover, excessive focus on the host ramifications of one specific bacterium may be misleading, as this neglects the synergistic action of bacterial communities (Lo et al., 2022). Bioreactors can be used to propogate and establish complex microbial communities by recapitulating the physiological conditions found in the GI tract (Guzman‐Rodriguez et al., 2018). In the context of exercise gut microbiota research, bioreactors may be used to identify bacterial communities responsible for specific, exercise‐related metabolites. Additionally, the potential for exercise‐mimetic dietary compounds (e.g., probiotics) to yield gut microbiota alterations that are similar to the beneficial effects of exercise may also be explored.

## 4 CONCLUDING REMARKS

Our understanding of exercise–microbiota interactions and subsequent implications for human health and performance has grown greatly over the past decade. Above, we provide an up‐to‐date summary of what is, to the best of our knowledge, all available literature that has characterized acute exercise responses of the gut microbiota and faecal metabolites in humans. In totality (Figure 1), these studies suggest that acute aerobic exercise has: (i) limited effects on intra‐ (α) and interindividual (β) diversity of the gut microbiota that may be more distinguishable with acute bouts of prolonged/extreme exercise, (ii) variable effects on specific microbial taxa that are likely a function of heterogeneity in exercise stimuli and interindividual factors, as well as methodological differences for microbiota measurements, and (iii) numerous effects on the metabolic activity of gut microbes with possible implications for host health and performance. Notably, though all‐inclusive, the above summary was drawn from only 11 studies (<200 participants), and there is still much to learn regarding the influence of acute exercise on the gut microbiota. Continued advancement of this body of knowledge has the potential to yield breakthroughs in the fields of sports and clinical medicine, as exemplified by recent work demonstrating that the gut microbiota plays a critical role in the efficacy of exercise for diabetes prevention (Liu et al., 2020).

Future research in this area is needed including well‐controlled (e.g., control group, multiple samples per time point, diet, etc.) laboratory studies of more heterogeneous participant populations (e.g., clinical populations and older adults), and considering the influence of different exercise modes (e.g., resistance exercise), doses and environmental stimuli (e.g., heat and altitude) on gut microbiota responses to acute exercise. Such studies may also aid in the understanding of the underlying mechanism(s) (e.g., increased gut motility, changes in blood flow or temperature, hormonal alterations, etc.) through which acute exercise influences the gut microbiota. This work will contribute to our understanding of the range of extraorganismal responses that underlie the effects of exercise, and will lay the foundation for strategic, evidence‐based exercise strategies to target the gut microbiota to improve health and performance.

## AUTHOR CONTRIBUTIONS

All authors read and approved the final version of this manuscript and agree to be accountable for all aspects of the work in ensuring that questions related to the accuracy or integrity of any part of the work are appropriately investigated and resolved. All persons designated as authors qualify for authorship, and all those who quality for authorship are listed.

## CONFLICT OF INTEREST

G.J.G. is a paid consultant for Compound Solutions, LLC. A.J.G. is a paid scientific advisor for Momentous and XPT Life, is a founder of RAPID Health Optimization, Vitality Blueprint, and Absolute Rest, and is on the board of directors for the Health and Human Performance Foundation.

## FUNDING INFORMATION

No funding was received for this work.
